# The enactment of physician-authors in Nobel Prize nominations

**DOI:** 10.1371/journal.pone.0242498

**Published:** 2020-11-23

**Authors:** Nils Hansson, Peter M. Nilsson, Heiner Fangerau, Jonatan Wistrand

**Affiliations:** 1 Department for the History, Philosophy, and Ethics of Medicine, Heinrich-Heine-University Duesseldorf, Duesseldorf, Germany; 2 The Unit for History of Medicine, Lund University, Lund, Sweden; Hackensack University Medical Center, UNITED STATES

## Abstract

Several physicians have been nominated for the Nobel Prize in literature, but so far none of them have received it. Because physicians as women and men of letters have been a major topic of feuilletons, seminars and books for many years, questions arise to what extent medicine was a topic in the proposals for the Nobel Prize and in the Nobel jury evaluations: how were the nominees enacted (or not) as physicians, and why were none of them awarded? Drawing on nomination letters and evaluations by the Nobel committee for literature collected in the archive of the Swedish Academy in Stockholm, this article offers a first overview of nominated physician-author candidates. The focus is on the Austrian historian of medicine Max Neuburger (1868–1955), the German novelist Hans Carossa (1878–1956), and the German poet Gottfried Benn (1886–1956), but it also briefly takes further physician-author nominees into account such as Sigmund Freud (1856–1939) and William Somerset Maugham (1874–1965). The article is part of an interdisciplinary medical humanities project that analyses nominations and committee reports for physicians and natural scientists nominated for the Nobel Prize from 1901 to 1970.

## Introduction

Physician-authors have long attracted the interest of scholars in the field of medical humanities. Why do they write? The reasons for the decision to pursue a literary career parallel to their medical work are of course multifaceted. As Anne Hunsaker Hawkins notes "the act of writing in some way seems to facilitate recovery: the healing of the whole person" [1], for patients as well as for the physicians. The sociologist Arthur Frank has concentrated on the question how illness "calls for stories" [2]. Illness requires stories in order to be understood and interpreted as part of a larger life story. This might not only be true for patients but also for the doctors who are exposed to their patients suffering in their daily practice [3]. Storytelling, in other words, is a part of the physician-author strategies as they seek to incorporate the experiences of their patients in a comprehensible setting.

Extensive lists of well-known and less famous physician-authors exist, made available by associations founded especially by and for physician-authors, like *The International Federation of Societies of Physician-Writers (FISEM)*, in 1973 renamed World Union of Physician Writers (UMEM). A closer look at these lists reveals that no physician-author to date has received the Nobel Prize in Literature so far. Does this matter? At least it does in terms of reputation for single authors but also for the group of physician-authors.

The aura of excellence surrounding the Nobel Prize is unparalleled [4]. As James F. English pointed out in his landmark study *The Economy of Prestige*: *Prizes*, *Awards*, *and the Circulation of Cultural Value*, the decisions by the Nobel committee not only means a commercial boom for the selected authors and their publishers, they also shape the literary canon globally [5]. Thus, it is not surprising that a growing number of scholars have described how the Nobel Prize has gained its scientific and cultural status in the public eye, and how the trophy has been used as a symbol to serve different agendas [6, 7].

The connection of the Nobel Prize’s aura with the lack of Nobel Prizes for physician-authors results in four questions:

- Which physician-authors were nominated for the Nobel Prize in Literature?
- To what extent was medicine a topic in the proposals and evaluations?
- Were the nominees, by nominators and/or Nobel committee members, enacted as physicians, and if yes, for what purpose?
- Why were the physician-authors in question finally not awarded?

In order to answer the proposed questions this article, stemming from an interdisciplinary medical humanities project that traces nominations and Nobel committee reports for physicians and natural scientists nominated for the Nobel Prize from 1901 to 1970, takes a closer look at physician-authors nominated for the Nobel Prize in literature.

### State of research

The Swedish innovator Alfred Nobel’s (1833–1896) will, which still is the core of the Nobel venture, stipulated that the literature prize was intended for an author who had produced “the most outstanding work in an ideal direction” [8]. Given the strong reputation of the award, it is a delicate task to select a Nobel Prize laureate. The standing and future of the prize is dependent on the work of its judges. Lars Gyllensten (1921–2006), so far was the only trained physician in the Nobel committee for literature, became a member of the Swedish academy in 1966 and acted as permanent secretary of the Academy between 1977 and 1986 and as member of the Nobel committee from 1968 to 1987. In his memoirs, published in 2000, Gyllensten reflected on the work in the committee: „Of course, there is a subjective element in every evaluation, in science as well as in literature and other aesthetic fields, but this is more obvious in the aesthetic field”[9].

Several researchers have dealt with the ‘hidden tracks’ in Nobel history by analyzing Nobel Prize nominations and Nobel committee evaluations with different research questions, ranging from uncovering networks between nominator and nominee [10] to reconstructing various ways of attributing excellence to scholars [11]. Scholars have used nominations and candidate evaluations to shed light on Nobel committee discussions behind the curtains, most notably Kjell Espmark’s overview of the criteria behind the jury decisions [12] and Bo Svensén’s two-volume compendium of Nobel committee evaluations [13, 14]. Since both of the authors have worked in or near the academy (Espmark was chairman of the Nobel committee in 1988, Svensén worked for the Secretary of the Swedish Academy between 1988 and 2006), they have detailed information about the prize procedure from an inside perspective, and at the same time they therefore may have a tendency to justify the Nobel committee decisions. Although Espmark and Svensén mentioned some of the authors to be discussed below in their books, they did not focus on the links to medicine.

## Material and methods

For this study we have analyzed nominations and committee reports for physicians nominated for the Nobel Prize in literature. Inclusion criteria were that nominees had a degree in medicine and that they had been nominated at least once. All nominations and evaluations for the Nobel Prize in literature are kept in the archive of the Swedish Academy in Stockholm, Sweden. Researchers can apply to study the files with the limitation of a 50-year embargo, meaning that in 2020 the documents between 1900 (first Nobel Prize nominations, the first prize was awarded in 1901) and 1970 may be accessed.

Language, length and style vary: Most of the nominations are written in English, German, French or one of the Scandinavian languages. Some contain only a few sentences, but in other cases nominators write 50–100 hundred pages in order to try to convince the jury. The latter is not seldom the result of campaigns where a comprehensive nomination is followed by numerous endorsement letters. One example is the nomination for the nowadays largely forgotten German author and physician Georg Bonne (1859–1945). The first nomination of Bonne, author of decidedly anti-Semitic books like *Der ewige Jude*. *Eine Menscheitstragödie* (1942) [The eternal Jew. A tragedy of humankind] or works like *Das Verbrechen als Krankheit* (1927) [The Crime as Disease] or *Über Eugenik und Euthanasie im Licht der nationalsozialistischen Ethik* (1934) [About eugenics and euthanasia in the light of National Socialist ethics], was signed by twelve scholars in 1926.

The method of selecting a Nobel laureate is quite similar in all of the five different prize committees (physics, chemistry, physiology or medicine, literature, and peace), but there is at least one important difference relevant for this study. In contrast to the Nobel science and medicine prizes, for which only former laureates as well as professors from a number of invited universities each year are eligible to nominate, all university professors of literature and philology as well as presidents of national literary societies may nominate candidates for the Nobel Prize in Literature. Once the nominations have been submitted, the proposals are discussed in the Nobel committee, and the most promising candidates are examined in detail. The Nobel committee evaluations were during the examined period of time written in Swedish.

Among the large pool of nominees (often 50–100 candidates per year), we find world-renowned candidates, but also–even in their time–completely unknown authors. After a systematical review of the Nobel nomination database for the Nobel Prize in literature (Nobelprize.org), we found thirteen nominated physician-authors from 1901 to 1969. Afterwards, the application (by the first author NH) to study the original documents at the Swedish Academy in Stockholm was accepted.

## Results

The scholars taken into account are listed in Table 1.

**Table 1:** Physician-authors nominated for the Nobel Prize.

Nobel Prize Nominee (Literature)	Citizenship	Specialty	Nomination year(s)
Max Neuburger (1868–1955)	Austria	Medical history	1924
Hans Carossa (1878–1956)	Germany	General practice	1942, 1949, 1950, 1952, 1955, 1956
Gottfried Benn (1886–1956)	Germany	Pathology, venerology	1953, 1954, 1955, 1956
Axel Munthe (1857–1949)	Sweden	General practice	1932
Georg Bonne (1859–1945)	Germany	General practice	1926, 1928, 1929, 1930, 1931, 1932, 1933
Sigmund Freud (1856–1939)	Austria	Neurology	1936
Karl Schönherr (1867–1943)	Austria	General practice	1911, 1912, 1933, 1938
Carl Gustav Jung (1875–1961)	Switzerland	Psychiatry	1954
Karl Jaspers (1883–1969)	Germany	Psychiatry	1950, 1960
Albert Schweitzer (1875–1965)	France	General practice, mission doctor	1952
William Somerset Maugham (1874–1965)	United Kingdom	Never practiced medicine	1955, 1959, 1961, 1962, 1964, 1965
Georges Duhamel (1884–1966)	France	Surgery	1936, 1937, 1940, 1942–1951, 1955–1957, 1961
Simon Vestdijk (1898–1971)	The Netherlands	Worked only few years as physician	1950, 1952, 1955, 1957, 1958, 1960–1962, 1964–1966

These names are, of course, not to be viewed as a "Who’s who"-list among physician-writers during the first seven decades of the 20th century. Several still today famous physician-authors like Anton Chekhov (1860–1904), Mikhail Bulgakov (1891–1940), Arthur Schnitzler (1862–1931), Louis-Ferdinand Céline (1894–1961), or William Carlos Williams (1883–1963) were never proposed. However, this sample of physician-authors reflects the wider picture of the "Nobel population" (both Nobel nominees and Nobel laureates) in the sense that men from the West (in this case only Europeans) are overrepresented. We found no female physicians among the nominees in the Nobel committee archive for literature. The only writer who had studied medicine (but never graduated) and subsequently received the Nobel Prize in Literature was Johannes V. Jensen in 1944 with the prize motivation: "for the rare strength and fertility of his poetic imagination with which is combined an intellectual curiosity of wide scope and a bold, freshly creative style."

In the following, we will in a chronological order briefly discuss the nominations and evaluations of Max Neuburger, Gottfried Benn, and Hans Carossa to show how their background and experiences in medicine colored the discussions in three different ways.

### The boundaries of literature? "Dull" medical history and Max Neuburger in 1924

**Fig 1:**
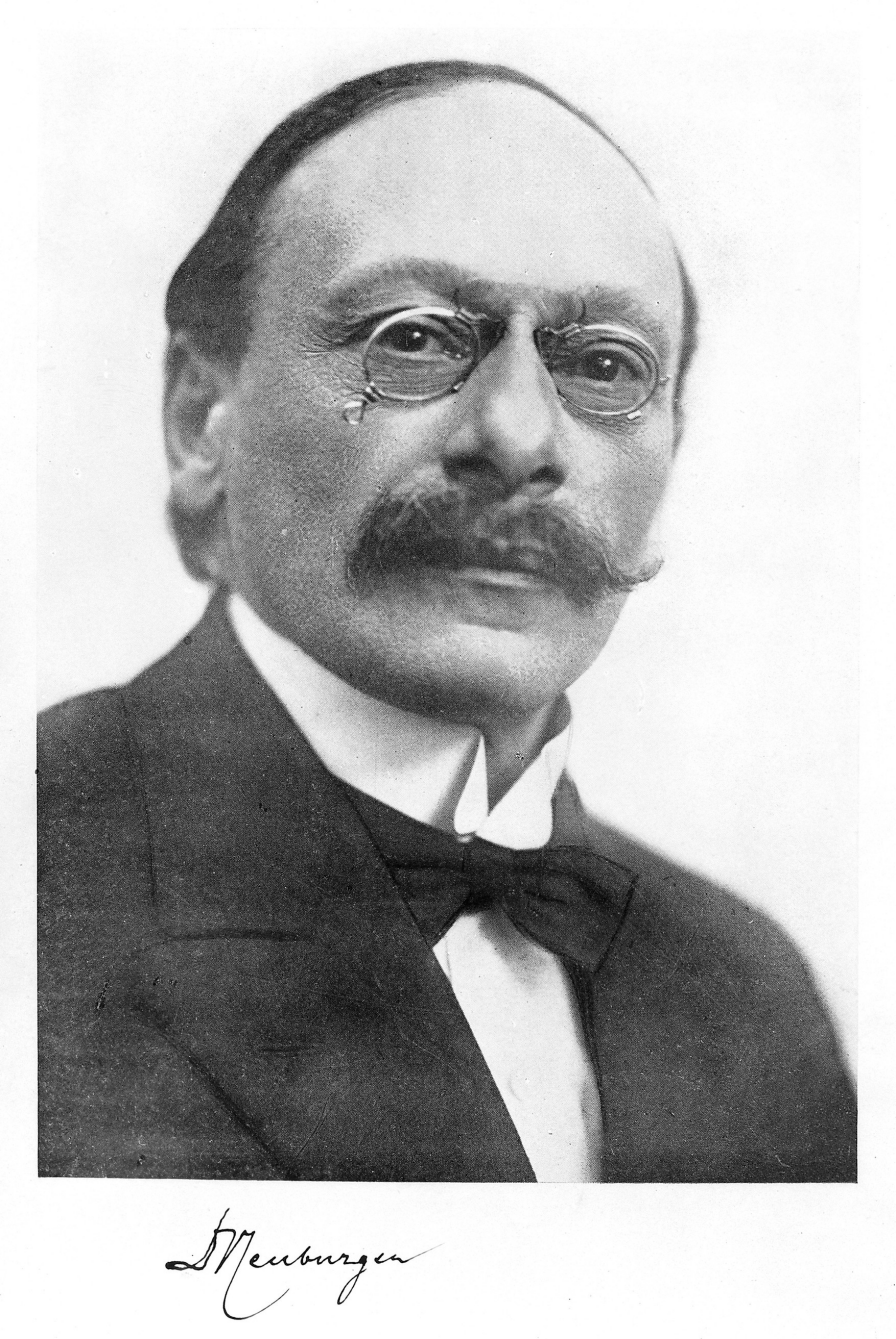
Portrait of Max Neuburger. Credit: Wellcome Collection. Attribution 4.0 International (CC BY 4.0).

The Austrian physician and medical historian Max Neuburger kept a private clinic until 1914 and was then appointed full professor in medical history in 1917 (Fig 1). Two years later he founded a medical history department at the *Josephinum* in Vienna. In 1938, he was forced to leave Austria because of his Jewish descent [15] and spent nine years at the Wellcome Historical Medical Museum in London, before he left for Buffalo (New York), United States. Neuburger returned to Vienna in 1952 and died there in 1955. Contemporary historians underline that Neuburger was as one of the internationally leading protagonists in the professionalization of medical history during the first half of the 20th century [16]. Still today a *Max Neuburger lecture* in honor of him is held at the *Josephinum* in Vienna.

In 1919, Neuburger nominated his colleague the medical historian Karl Sudhoff (1853–1938) for the Nobel Prize, but not for the literature Prize. He proposed him in the category physiology or medicine [17]. The nomination did not bear fruit. Was it perhaps a wiser strategy to propose historians of medicine for the literature prize?

The Norwegian physician, orientalist and medical historian Adolf Fonahn (1873–1940) nominated Neuburger for the literature prize in 1924. He argued that the prize already had been awarded to “scientific authors” and that Neuburger, therefore, was a possible candidate (perhaps he meant the 1902 prize to the historian Theodor Mommsen (1817–1903), "the greatest living master of the art of historical writing, with special reference to his monumental work, *A history of Rome*", as the Nobel jury characterized him in the prize motivation). Neuburger had according to Fonahn succeeded in making medical history a “serious academic discipline”. Neuburger would thus remain a "giant" in the field, and the importance of his book *Geschichte der Medizin* was "monumental". Fonahn attached several publications by Neuburger to the nomination dossier.

Per Hallström (1866–1960), member of the Swedish Academy from 1908 to 1960, evaluated Neuburger the same year. He acknowledged that Neuburger had published several studies ("some with interest for the humanities: *Schillers Beziehung zur Medizin*") and that he had received numerous honors from foreign scientific societies. Hallström chose to focus on his opus magnum: *Geschichte der Medizin*, published 1906–1911 and noted that such textbooks must be of interest to a wider audience than specialists to be considered for a prize. According to Hallström, Neuburger’s book indeed had such qualities, "his cultural history approach" had in his view been communicated in a "concise, clear, and thought-provoking manner". That said, Hallström concluded that the main purpose of the book was to explain and contextualize medical facts, and that it is " a bit dull", a deadly blow against a candidate. Therefore, he concluded that Neuburger should not be taken into closer consideration.

### Medicine, politics and poetry: The nominations for Gottfried Benn

In the 1950s, the pathologist, venerologist and poet Gottfried Benn was nominated five times for the Nobel Prize in Literature [18]. The first nomination was submitted by the German-Austrian linguist Heinrich Kuen (1899–1989) in 1953. In his brief letter, Kuen wrote that Benn's poetry was "world literature" and added that he was as an important representative of the vibrant Berlin literary scene. Ernst Alker (1895–1972), Austrian-Swedish literary scholar, posted a more detailed motivation in favor of Benn, containing the punchline: "[He is] the only one among the living German poets who has gained an international reputation."

Alker also admitted that Benn’s collaboration with the National Socialist Party during the 1930s certainly was an aggravating circumstance (e.g. he was the Chairman of the Poetry Section in the Reich Chamber of Culture) [19]. However, that issue was only once brought up in the Nobel Committee protocols. In a Benn evaluation, the Swedish poet Bertil Malmberg (1889–1958) meant that the committee should look beyond Benn’s early connections to the Nazi party and instead focus on his poetry.

Much is written about how Benn’s experiences in hospitals colored his writings, apparent already in his first book entitled *Morgue und andere Gedichte* (Morgue and other poems), published in 1912 while he worked as assistant pathologist. Benn’s work as pathologist and venerologist was repeatedly brought up in the nominations. Alker argued that it was a "proof of his intellectual capacity". In a 16-page nomination letter, the Swedish poet Bertil Malmberg submitted a comprehensive proposal for Benn in 1953. He argued that the influences from “laboratories and dissecting rooms, from clinics and hospitals, from bacteriology […] created waves that had hit the literature”, and that Gottfried Benn was a central character in this context. He had according to Malmberg succeeded in transforming his experiences as young doctor to compelling poems, novels and essays.

In spite of the strong support, the nominators did not convince the Nobel jury. Benn was in the end not viewed as prize-worthy. What Benn himself thought about the prize is unclear, however, even before his first nomination he wrote in February 1949 to the Bremen merchant Friedrich Wilhelm Oelze (1891–1978), who had mentioned him in a previous letter as a possible candidate: "Nobel Prize. No jokes, please! I know where I belong and where not" [20].

Even after his death in 1956, Benn was mentioned in the Nobel files. In 1961, the Stuttgart professor of Literature Fritz Martini (1909–1991) wrote in a nomination that Benn along with Bertolt Brecht (1898–1956) would have been obvious candidates, but since they both had passed away and posthumous prizes were not allowed, he put forward Heinrich Böll (1917–1986), who was to receive the award in 1972.

### "He spent most of life caring for patients": Hans Carossa as Nobel nominee

Hans Carossa is one of the most well-known German physician-authors of the 20 Century [21]. Several schools and streets in Germany are named after Carossa and biographical sketches of him emphasize that several of his novels circle around medical topics and the patient-physician relationships [22]. This is also apparent in some of his book titles like "Doktor Bürgers Ende" (1913), "Der Arzt Gion" (1931), and "Der Tag des jungen Arztes" (1955).

Carossa studied medicine in Munich and Wurzburg and earned the title Dr. med. at Leipzig university in 1903. One year later, he took over the medical practice of his father in the city of Passau in southern Germany close to the Czech border. In 1910, his first poems *Gesammelte Gedichte* were published. He received several awards and honors for his books, including the Gottfried Keller Prize (1931), the Goethe Prize (1938), and the Grand Cross of Merit of the Federal Republic of Germany (1953). On his 70th birthday, he was awarded a doctor honoris causa of the University of Munich.

From 1942 to 1956, Carossa was nominated at least six times [22]. In 1952, the Munich Professor of Modern German Literature Hans Borcherdt (1887–1964) wrote to the Nobel Committee that German poetry "is experiencing a crisis at the time", and that there are no "young German authors that are able to compete on an international scale". Therefore, Borchert wanted to nominate an author of "the same generation as Hermann Hesse and Thomas Mann […] I mean Hans Carossa". Borcherdt underlined the fact that medicine is a common topic in Carossa’s books: "Three of his novels circle around the life of physicians", additionally "Carossa dedicated most of his life to patients […] as a lung disease specialist. It is characteristic of his nature that he has always kept his distance from the great centers of modern civilization, preferring to practice his practice in villages. Thus, he lived as a human being, as he proclaimed as a poet".

That year, Carossa was evaluated by Per Hallström, who judged him as prizeworthy, but the committee preferred other candidates. As was the case with Gottfried Benn, Carossa was repeatedly discussed in the prize committee and viewed to be a strong nominee. In 1955, one year before he passed away, the committee stated that with the book "Der Tag des jungen Arztes" he has continued his very "captivating autobiography" that " most certainly will become one of the classical novels in the literary canon of modern Germany". Nevertheless, the committee concluded that it could not yet give him a full recommendation [23].

## Discussion: All Nobel laureates are alike; each unhappy nominee is unhappy in its own way

The result of this overview is not clear-cut. Some of the nomination letters did barely contain any links to medicine at all. In the proposals for William Somerset Maugham, for instance, it was only with the abbreviations M.R.C.S. and L.R.C.P. indicated that he was a member of the Royal College of Surgeons (England) and a Licentiate of the Royal College of Physicians of England. Medicine was not a focus in the nominations for the Dutch physician Simon Vestdijk either [24]. Other nominators emphasized that the works by the nominee in question were closely intertwined with medicine, as was the case with Gottfried Benn and Hans Carossa.

The nomination for Neuburger, in particular, raises the question what is to be considered as *literature* and what as *medicine* in a Nobel Prize context. The Nobel committee seems not to have had a clear definition, and the boundaries have changed over time. The topic was repeatedly discussed in the committee, e.g. after nominations of physicians who had been nominated for a Nobel Prize in more than one category. When Sigmund Freud was proposed for the literature prize, for instance, the committee members stated that he should better have been nominated for the prize in physiology or medicine [25]. (In fact, Freud had also been proposed for the physiology or medicine prize, no less than 32 times from 1915 to 1938 for his work on psychoanalysis and its treatment methods). The same is true for the nomination for Carl Gustav Jung in 1954, although the nominator Alker emphasized that "the nomination does not refer to the famous physician and psychiatrist Jung, but rather his work that has largely influenced the humanities". Nevertheless, the jury meant that “the nomination for the literature prize must have been a mistake”. Similarly, the Nobel committee for literature admired the philosopher and psychiatrist Karl Jaspers for his publications, but it did not believe a full evaluation would be necessary at the time. The committee stated that his achievements could not–from a ‘Nobel’ perspective–be characterized as literature "in an ideal direction" (Nobel committee statement in 1960). The case of the physician and philosopher and Albert Schweitzer is different. He was praised by committee member Hallström as both an important writer and as a great personality, but since the committee knew that he in 1952 next to the literature prize also has been nominated for the Peace Prize (which he was to receive) they decided to postpone the final decision.

Nobel Prize nominations and evaluations give new insights into the reputation of some physician-authors of the 20th century, particularly regarding the nominees who never received the award, or to paraphrase the Anna Karenina-principle: "All Nobel laureates are alike; each unhappy nominee is unhappy in its own way". Most of the above-mentioned doctors (Table 1) gave up their work as physicians to write. Although they were portrayed primarily as poets, novelists, historians or philosophers, several of them were enacted as physicians as well. Nominators stressed that their experiences as doctors not only strongly influenced their writing but also made them more trustworthy when they described suffering, poverty, and the relationship between physician and patient. In one case (Benn), the medical degree was used as a sign of intellectual capacity. Further research shall analyze the nominations and evaluations of other physicians and natural scientist-authors to look at similarities and differences in how science and medicine were used as symbols in the proposals for the Nobel Prize in literature.
